# *COQ2* mutation associated isolated nephropathy in two siblings from a Chinese pedigree

**DOI:** 10.1080/0886022X.2020.1864402

**Published:** 2021-01-05

**Authors:** Min Li, Zhihui Yue, Hongrong Lin, Haiyan Wang, Huamu Chen, Liangzhong Sun

**Affiliations:** a Department of Pediatrics, Nanfang Hospital, Southern Medical University, Guangzhou, China; b Department of Pediatrics, The First Affiliated Hospital, Sun Yat-sen University, Guangzhou, China; c Department of Pediatrics, Sun Yat-sen Memorial Hospital, Sun Yat-sen University, Guangzhou, China

**Keywords:** CoQ10, steroid-resistant nephrotic syndrome, mitochondria, mutation, child

## Abstract

**Backgroud:**

Coenzyme Q10 (CoQ10) is involved in the biosynthesis of adenosine triphosphate (ATP), and is most abundant in the mitochondrial membrane. The primary CoQ10 deficiency caused by *COQ2* defect is mostly manifested as encephalopathy, encephalopathy with nephropathy, and rarely as an isolated nephrotic syndrome.

**Methods:**

Clinical and pathological data and peripheral blood samples of 2 siblings with steroid-resistant nephrotic syndrome (SRNS) and their family members of a Chinese pedigree were collected. DNA was extracted and subjected to next-generation sequencing of target genes of hereditary nephropathy.

**Results:**

Compound heterozygous mutations of *COQ2* (c.1058A > G, p.Y353C, paternal and c.973A > G, p.T325A, maternal)were identified in both siblings of the pedigree. Mutation of p.Y353C was novel. The proband was a girl, who presented with SRNS at the age of 7 months. CoQ10 was administered after the gene sequencing results came out. Proteinuria decreased gradually to 1+, occasionally negative. The child was normal in growth and intelligence. She is now 4 years old. The second patient was her elder brother. He was found to have SRNS at the age of 2 years old. Renal pathology indicated focal segmental glomerulosclerosis (FSGS). Electronic microcopy revealed that a large quantity of mitochondria with normal contour was accumulated within the podocytes. Both patients were in normal intelligence without convulsion.

**Conclusion:**

The 2 cases harboring *COQ2*compound heterozygous mutations presented with isolated SRNS, with a renal pathology of FSGS and a large quantity of mitochondria with normal contour accumulated within the podocytes. CoQ10 was efficacy in eliminating proteinuria.

Coenzyme Q10 (CoQ10), the electron carrier of the respiratory chain, transmitting electrons of complex I and II to complex III, is involved in the biosynthesis of adenosine triphosphate (ATP), and is most abundant in the mitochondrial membrane. Intracellular synthesis is the main source of CoQ10 [1,2]. More than 13 genes have been found to be involved in the biosynthesis of CoQ10, including *COQ2* [3]. CoQ10 synthase-related gene defects can lead to CoQ10 deficiency in an autosomal recessive disorder and often involves multiple systems [1–3]. The primary CoQ10 deficiency caused by *COQ2* defect is mostly manifested as encephalopathy, encephalopathy with nephropathy, and rarely as an isolated nephrotic syndrome [3]. Here, we presented 2 cases with isolated steroid-resistant nephrotic syndrome (SRNS) who harbored compound heterozygous *COQ2* mutations in a Chinese family.

## Materials and methods

The study was performed in accordance with the Declaration of Helsinki and was approved by the ethics committee of the First Affiliated Hospital, Sun Yat-sen University (Number: [2011]17) Written informed consent was obtained from their parents and control subjects. Clinical and pathological data were collected from the patients and their family members. Blood samples of the patients and their family members were collected.

## Targeted exome sequencing (TES) and bioinformatics analysis

Genomic DNAs were extracted from peripheral blood samples. TES was carried out in the proband of the pedigree using a gene panel comprising 250 known genes associated nephropathy. The minimum average sequencing depth was 150×. Candidate variants identified by TES were further confirmed by Sanger sequencing. The confirmed variants were then checked in the genomic DNAs of the family members. The detection was carried out by Beijing Mygenostics Co., LTD, Beijing, P. R. China. Clustal X was used for alignments, and Predict Protein (https://predictprotein.org) was used to identify the conservation of novel mutated amino acid residues and for predicting the secondary structure of novel missense mutations. The Combined Annotation Dependent Depletion (CADD) score was used for the evaluation of missense mutations.

## Results

### Clinical and pathological data

**Figure 1.**
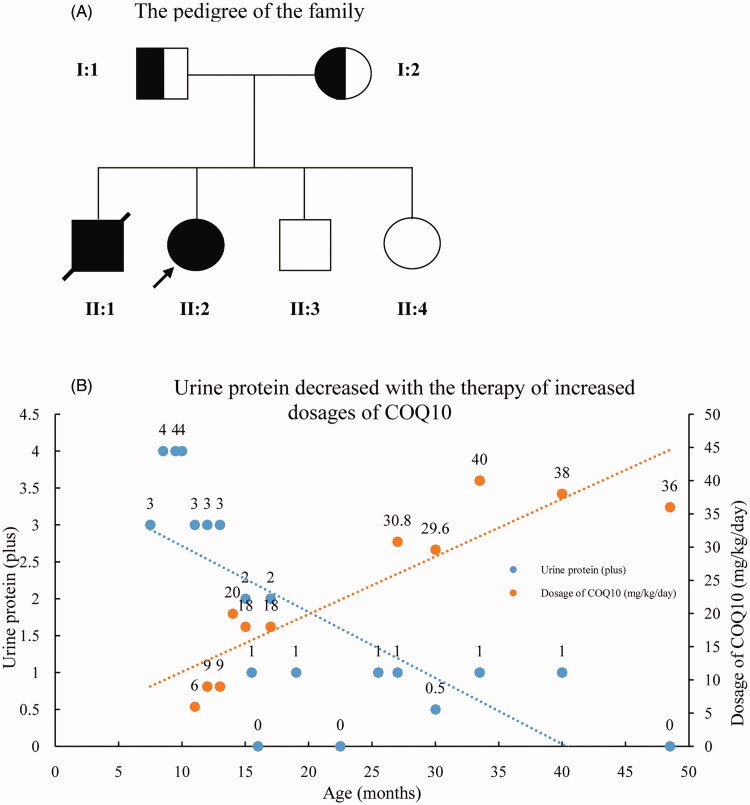
The pedigree chart of the family and relationship between dose of CoQ10 and urinary protein. (A) Pedigree chart of the family. The filled circle denotes the proband, and the filled square donates her died brother. (B) The patient’s urinary protein decreased with the increasing dose of CoQ10.

**Figure 2.**
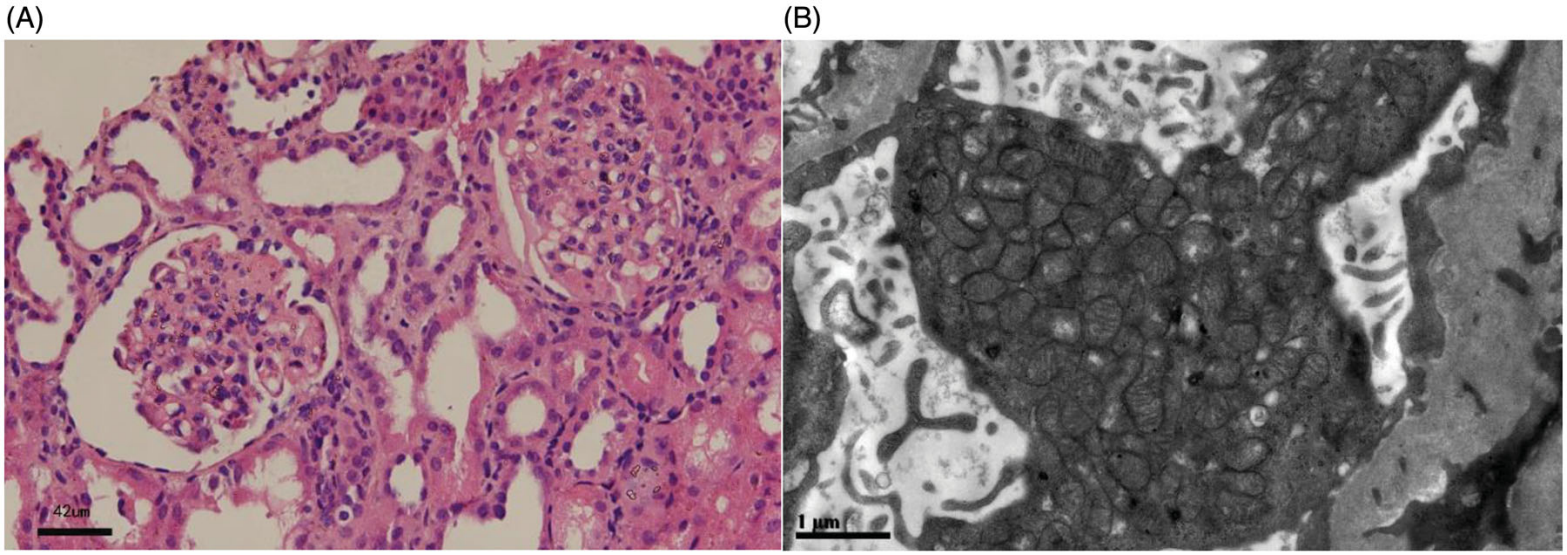
Renal pathological changes of the proband’s elder brother harbored *COQ2* mutations. (A) The glomeruli showed segmental sclerosis and adhesion to the Bowman’s capsule wall (Light microscope, hematoxylin eosin staining, Scale bar: 42 μm). (B) Mitochondria accumulated in the podocyte with normal contour (Electron microscope, Scale bar: 1 μm).

The pedigree was Chinese Han ethnicity, originated from Shantou, Guangdong, China. Both the parents were healthy and had a non-consanguineous marriage (pedigree chart, Figure 1(A)). They had 4 children. The proband was a girl, who was found to have edema and proteinuria at the age of 7 months. Serum albumin was 9.1 g/L, cholesterol 15.2 mmol/L, triglyceride 2.18 mmol/L, alanine aminotransferase 30 U/L, aspartate aminotransferase 45 U/L, glucose 4.3 mmol/L, carbon dioxide 24 mmol/L, urea nitrogen 3.2 mmol/L, creatinine 24 μmol/L, uric acid 175 μmol/L, anion gap 13 mmol/L. Steroids had been tried for 8 weeks without effect. Steroids withdrawn gradually and Benazepril was administered with a daily dose of 2.5 mg. Proteinuria didn’t drop down apparently. Renal biopsy was not accepted. An association between CoQ2 mutation and renal disease was implied according to the genetic sequencing results. CoQ10 (from 3 mg/kg increased to 10 mg/kg, 3 times a day) was administered at the age of 11 months, and proteinuria decreased gradually to 1+ positive (Figure 1(B)), occasionally negative, and serum albumin increased to 38 g/L. The child was normal in growth and intelligence. She is now 4 years old. CoQ10 is now given with a dose of 200 mg, three times a day. Proteinuria was ranged from negative to 1+ positive; urine albumin was 65.8 mg/L. Serum albumin was 39.6 g/L and serum creatinine was 26 μmol/L. The second patient was the elder brother of the proband. He was found to have SRNS at the age of 2 years old. Serum albumin was 10.8 g/L, and cholesterol 9.3 mmol/L. Renal pathology indicated focal segmental glomerulosclerosis (FSGS) (Figure 2(A)). Electronic microcopy revealed that a large quantity of mitochondria with normal contour was accumulated within the podocytes (Figure 2(B)). The child progressed to ESRD at the age of 5 years old. Renal replacement therapy was not accepted and he was taken back to his hometown by his parents and he died 2 weeks later. There was no extra-renal manifestation, and genetic results were not available before his death.

### Genetic sequence results and bioinformatic analysis

**Figure 3.**
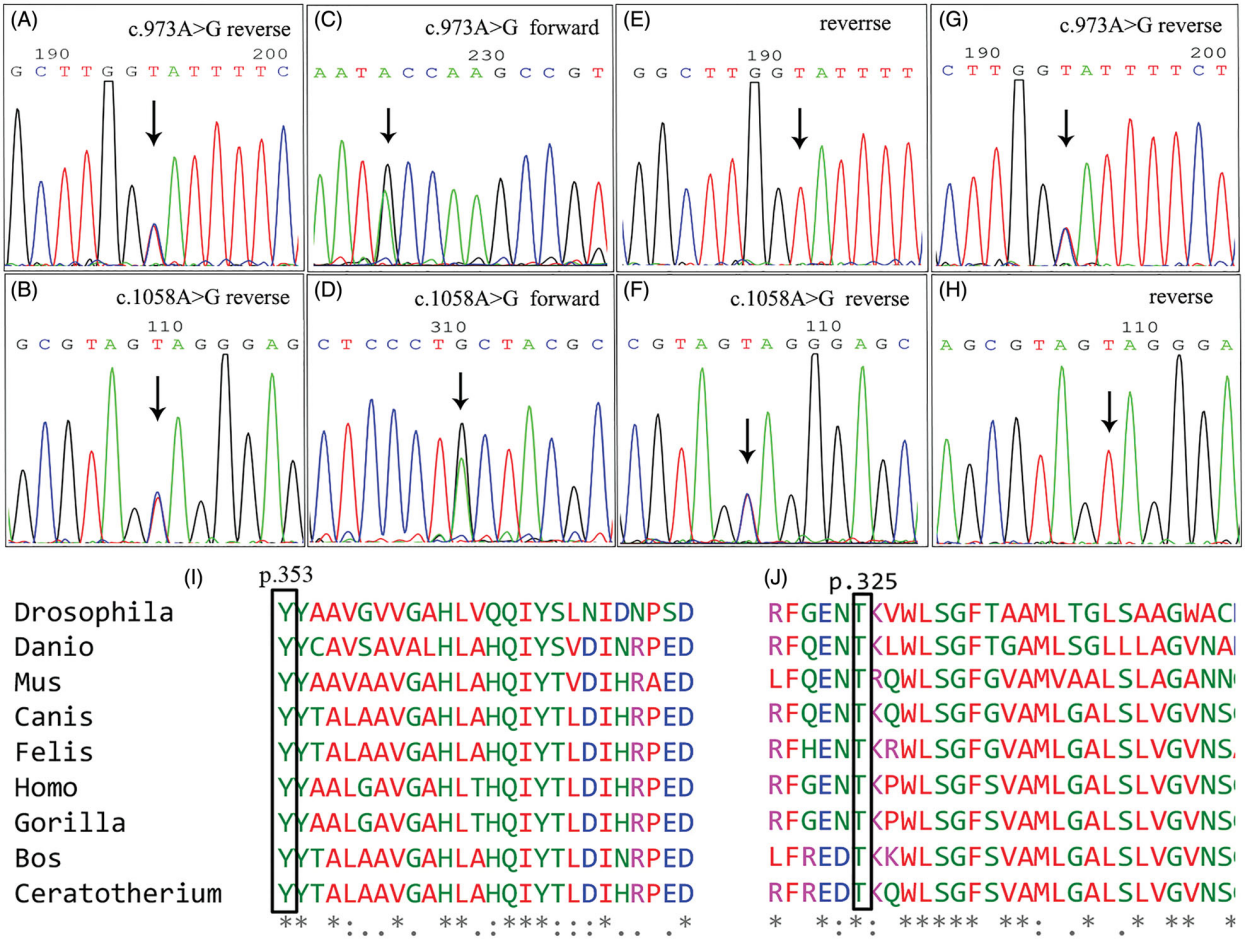
The identified *COQ2* mutations of the pedigree. A and B: proband; C and D: elder brother of the proband; E and F: the father; G and H: the mother. I and J: Multiple alignment of amino acid sequences across species using Clustal X, which showed high conservation of the amino acids at locus 353 and locus 325 of COQ2.

TES of the proband revealed compound heterozygous missense mutations of *COQ2* (c.1058A > G, p.Y353C and c.973A > G, p.T325A). Her elder brother harbored the same mutations as she did. Both mutations of *COQ2* were inherited from their parents, respectively[c.1058A > G, p.Y353C was inherited from the father and c.973A > G, p.T325A was inherited from the mother (Figure 3(A–H))]. The c.1058A > G mutation was paternal, causing the change of tyrosine to cystine at the amino acid site of 353. The c.973A > G mutation was maternal, causing the change of threonine to alanine at the amino acid site of 325. Both amino acids at the mutant loci were highly preservative during evolution (Figure 3(I–J)). The CADD scores of the 2 mutations were 25.4 and 24.7, respectively, and were predicted to be damaging. Mutations in other genes related to CoQ10 deficiency were not found

## Discussion

The clinical manifestations of CoQ10 deficiency caused by different biosynthetic enzyme defects and the affected organs are not consistent, and different clinical phenotypes can be caused by the same synthetic enzyme deficiency [4]. Three groups of CoQ10 synthetic enzyme deficiencies were proposed by Acosta et al. [3]. The first group includes *PDSS1, PDSS2, COQ2, COQ6,* and *ADCK4*. Glomerular involvement manifests as SRNS, is a clinical feature that maybe present in the defects of these genes, together with or without neurological or systemic disorders. The second group encompasses *COQ4, COQ7,* and *COQ9*. The defects of these genes mainly manifest as encephalomyopathy. Glomerular impairment has never been displayed, although tubulopathy may be present. The third is the only pathogenic gene of *ADCK3*. Central nervous system (CNS) involvement is essential. Extra-CNS impairment has not been observed [5].

Approximately 1% of primary steroid-resistant nephrotic syndrome (SRNS) patients were affected by genetic defects in CoQ10 synthase [6]. *COQ2* is the first pathogenic gene identified in association with CoQ10 deficiency [7]. The onset of nephropathy usually occurs in infants and young children, but it can also appear as late as during puberty [8,9]. Once the kidney disease develops, the disease would progress rapidly without treating appropriately. Renal pathology was primarily FSGS. Extensive podocyte foot process effacement and accumulation of deformed mitochondria in podocytes are common features under electron microscopy [7–9]. In the early stage of the disease, supplementation of CoQ10 can effectively alleviate clinical manifestations, but may not be sustained or completed [7–11].

In China, there has been only 1 case reported with renal involvement recently, in which an infantile case presented with SRNS at the age of 11 months, with mild motor development retardation and moderate language development retardation [12]. In the present study, both of the siblings presented with isolated SRNS. They harbored compound heterozygous mutations of *COQ2*. The mutation of p.Y353C was novel. The proband presented SRNS at the age of 7 months. Renal impairment was relieved with CoQ10 treatment. The patient was normal in growth and development and has been followed up for 45 months. The other 1 had SRNS at 2 years old and progressed to ESRD at the age of 5. Renal pathological change was FSGS. Accumulation of mitochondria in the podocytes was observed but showed normal contour with no obvious swelling or rupture. This is different from previous reports and warrants further research. Neither patient in this study experienced any involvement of extra-renal organs, such as the CNS.

The pathogenesis of CoQ10 deficiency has not been fully understood. Mutation of pathogenic genes eventually leads to a decrease in CoQ synthesis, affecting energy metabolism and bio-oxidation of cells, insufficient ATP production, and decreased antioxidant capacity [1–3]. *COQ2* is located at 4q21 and has 7 exons. The encoded protein is parahydroxybenzoate polyprenyl transferase, which catalyzes the prenylation of parahydroxybenzoate with a polyprenyl group [7,12]. Human COQ2 protein contains nine transmembrane domains, a cytoplasmic domain, and a non-cytoplasmic domain. The activity of the enzyme is mainly in the transmembrane domain and the non-cytoplasmic domain [13].The relationship between *COQ2* genotype and phenotypesis not very clear yet. Through the construction of yeast expression vector, Quinzii et al. [13] had found that there was a correlation between different levels of residual CoQ and the severity of clinical manifestations (such as encephalopathy). However, the relationship between nephropathy and its severity and CoQ production is not clear and maybe more complicated [13–15]. The mutant sites of *COQ2* related to nephrology were inconsistent, and mainly located within the I to the IV transmembrane domains in previous studies [14]. In the present study, two of the mutant sites were located within the VI to the VIII transmembrane domains. Therefore, the two mutations maybe impact the transmembrane function of COQ2.

In conclusion, the phenotype of CoQ10 deficiency caused by *COQ2* defects is discordance. Although isolated nephropathy is rare, CoQ10 deficiency should be considered in patients with primary SRNS, especially those whose renal pathology is FSGS and mitochondria gathering in podocytes. The present study also suggested that the mitochondria accumulated in podocytes could be normal in appearance.
